# Urology during Afghanistan mission: lessons learned and implications for the future

**DOI:** 10.1007/s00345-023-04475-z

**Published:** 2023-06-23

**Authors:** Justine Schoch, Cord Matthies, Holger Heidenreich, Jens Diehm, Hans Schmelz, Christian Ruf, Tim Nestler

**Affiliations:** 1 Department of Urology, Federal Armed Forces Hospital Koblenz, Koblenz, Germany; 2 Department of Urology, Federal Armed Forces Hospital Hamburg, Hamburg, Germany; 3 Department of Urology, Federal Armed Forces Hospital Berlin, Berlin, Germany; 4 Federal Armed Forces Hospital Koblenz, Koblenz, Germany; 5 Department of Urology, Federal Armed Forces Hospital Ulm, Um, Germany; 6 Department of Urology, Faculty of Medicine and University Hospital Cologne, University Cologne, Cologne, Germany

**Keywords:** Genitourinary injury, Battle-related injury, Urological surgery, Reconstructive surgery, Laparotomy

## Abstract

**Purpose:**

Battle-related trauma is common in modern warfare and can lead to genitourinary injuries. In Western countries, urogenital injuries are rare in the civilian environment. The main objective of this study was to assess urological workload for surgeons on deployment.

**Material and methods:**

Data were acquired over a period of five years of deployment in a U.S. facility in Afghanistan.

**Results:**

German urological surgeons treated on average one urologic outpatient per day and performed 314 surgical interventions overall. Surgical interventions were categorized as battle-related interventions (BRIs, n = 169, 53.8%) and nonbattle-related interventions (non-BRIs, n = 145, 46.2%). In the BRI group, interventions were mainly performed on the external genitalia (n = 67, 39.6%), while in the non-BRI group, endourological procedures predominated (n = 109). This is consistent with a higher rate of abdominal or pelvic procedures performed in the BRI group (n = 51, 30.2%).

Furthermore, the types of interventions performed on the external genitalia differed significantly. In the BRI group, 58.2% (n = 39) of interventions were scrotal explorations, but none of those procedures were performed in the non-BRI group (p < 0.001). However, 50.0% (n = 13) of scrotal explorations in the non-BRI group were due to suspected torsions of the testes followed by orchidopexy (BRI: n = 1, 1.5%, p < 0.001). Concerning outpatients, the consultation was mainly due to complaints concerning the external genitalia (32.7%, n = 252) or kidney/ureteral stones (23.5%, n = 181).

**Conclusion:**

While the treatment of urological outpatients in a deployment setting resembles the treatment of soldiers in Germany, BRIs requires abdominal/retroperitoneal urosurgical skills and basic skills in reconstructive surgery.

**Supplementary Information:**

The online version contains supplementary material available at 10.1007/s00345-023-04475-z.

## Introduction

In modern warfare, the mortality of battle-related injuries decreased to approximately 10% in Vietnam/Afghanistan compared to 30% in World War II [1]. Mainly due to the use of Kevlar body armor, not only the overall mortality but also the incidence of abdominal urological injuries was reduced in current conflicts such as Operation Desert Storm in Iraq, where kidney injuries appeared in 17% of combatants compared to a higher rate of 31% in the Vietnam war. A decrease in abdominal wounds due to improvement in body armor resulted in a relatively higher rate of injuries involving the external genitalia due to shrapnel injuries caused by explosive devices [2]. Commonly observed comorbidities to genitourinary injuries (GUIs) included lower extremity amputations, colorectal injuries or traumatic brain injuries (TBI) [3]. GUIs occur in approximately 5% of modern combat-related injuries and are usually treated with wound debridement and delayed fashion of soft tissue. The basis of wound coverage often involves the use of split-thickness skin grafts or local flaps. Complex final reconstruction is usually delayed [4].

Domestically, GUIs comprise a low percentage of traumatic injuries (2%) and are most likely associated with traffic accidents or sporting items in younger patients, while in older patients, GUIs are commonly associated with falls [5, 6]. While the mechanism of battle-related GUIs is commonly improvised explosive devices or blasts, blunt trauma with injuries of the kidney predominates domestically [7, 8]. Furthermore, in the treatment of domestic GUIs, organ salvage has become common due to advantages in imaging and minimally invasive techniques, and there are established guidelines for treating GUIs [9]. In contrast, there are no established guidelines for treating battle-related GUIs, but a few studies have pointed out the importance of wound debridement and reconstructive surgery [10, 11].

This study includes the only existing data concerning urologic diseases and injuries during the war in Afghanistan and the primary objective was to evaluate the urological case-load abroad and especially to identify the challenges for urological surgeons in modern deployments using the example of a deployed U.S. facility (Role 3) in Afghanistan concerning incidence and management of battle-related intervention. Furthermore, this study focused on the possible need for special training of urological surgeons in preparation for foreign assignments.

## Materials and methods

Prospective data acquisition was performed anonymously over a period of five years (26/04/2015 to 18/05/2020), excluding a year of missing data (20/09/2017 to 11/10/2018). All patients who were treated as outpatients by German urological surgeons in a deployed U.S. facility (Role 3) in Bagram, Afghanistan, or required urogenital surgical interventions were documented by the treating urological surgeon. The study was conducted in compliance with the Declaration of Helsinki (as revised in 2013). As no specific patient data were collected approval from the ethics committee was not required.

In this period of time, approximately 10.000–20.000 U.S.-American or allied soldiers were based at the Bagram air field and furthermore civilians, GO- and NGO members were also treated by German urologists in Bagram. To compare the distribution of the different disease patterns in urological outpatient care in Afghanistan with those in regular care in Germany, we evaluated disease patterns in the Military Armed Forces Hospital Koblenz in Germany over a period of two months (10/2022–12/2022) in the same categories as in mission.

Statistical analysis was performed using the IBM SPSS Statistics system for Windows (v24.0) (Armonk, NY, USA). Categorical variables are presented as n (%). Data were analyzed using Pearson’s chi-square test. Differences were considered statistically significant at p < 0.05.

## Results

**Fig. 1:**
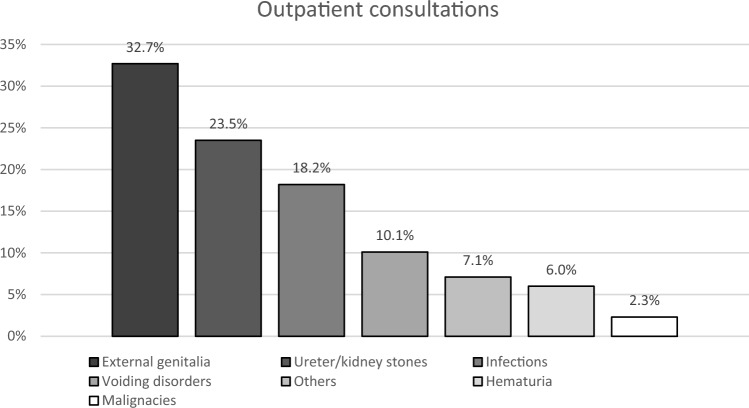
Outpatient consultations without reconsultations (n = 733). *External genitalia* summarizes pain or swelling of the external genitalia due to hydro/varicocele or hernia and meatus stenosis. OR procedures were excluded. Consultations of one patient could contain more than one diagnosis (n = 770)

In a period of four years in a deployed U.S. facility in Afghanistan, German urological surgeons treated 733 outpatients in 1011 outpatient consultations. On average, 0.7 patients were seen per day (range: 0–8 consultations per day). A single patient consultation could contain more than one diagnosis. Further analysis refers to the number of outpatient diagnoses without reconsultations. The main reasons for outpatient urological consultations were complaints concerning the external genitalia, such as unspecific pain, varicocele, hydrocele, trauma, meatus stenosis, hernia (n = 252, 32.7%), kidney/ureteral stones (n = 181, 23.5%) or infections (n = 140, 18.2%) (Fig. 1). Consultations due to voiding disorders (n = 78, 10.1%), hematuria (n = 46, 6.0%), and other complaints, such as unspecific back or abdominal pain (n = 55, 7.1%), were less frequent. The rate of suspected malignancies was low, but they were present (n = 18, 2.3%). To compare the distribution of disease patterns occurring on deployment, we analyzed disease patterns occurring in urological outpatient care in a domestic military hospital in Germany. In a 2-month period (10/2022–12/2022), 720 outpatients with 817 diagnoses were seen. On average, 15 patients were treated per day (range 1–25 consultations per day). Reconsultations were excluded from the study. The main reasons for consultations for urological outpatient care in Germany were medical screening and follow-up issues (n = 407, 49.8%). Other reasons for consultation that did not occur during deployment were erectile dysfunction (n = 40, 5%), testosterone substitution (n = 20, 2.4%), confirmed or suspected prostate cancer (n = 29, 3.5%) and follow-up of renal cysts (n = 18, 2.2%). These categories were excluded from further analysis, resulting in a total of n = 302 patients (Supp. Figure 1). Not only on deployment but also domestically, most patients presented themselves with complaints concerning the external genitalia (domestically: n = 87, 28.8%; on deployment: n = 252, 32.7%, p = 0.178). However, in Germany, the relative distribution of voiding disorders (domestically: n = 76, 25.1%; on deployment: n = 78, 10.1%, p < 0.001) and kidney/ureter stones (domestically: n = 26, 8.6%; on deployment: n = 181, 23.5%, p < 0.001) (Supp. Figure 2) differed significantly from the distribution of disease patterns on deployment (Fig. 1).

**Fig. 2:**
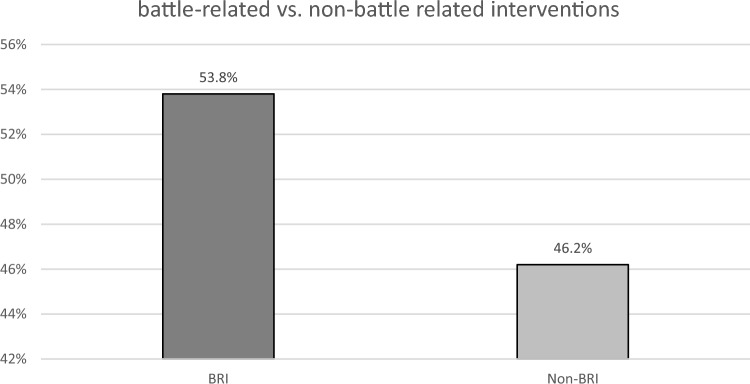
Urological surgical interventions (n = 314). Within urological interventions, 53.8% (n = 169) were considered battle related, while 46.2% (n = 145) were considered nonbattle related

**Fig. 3:**
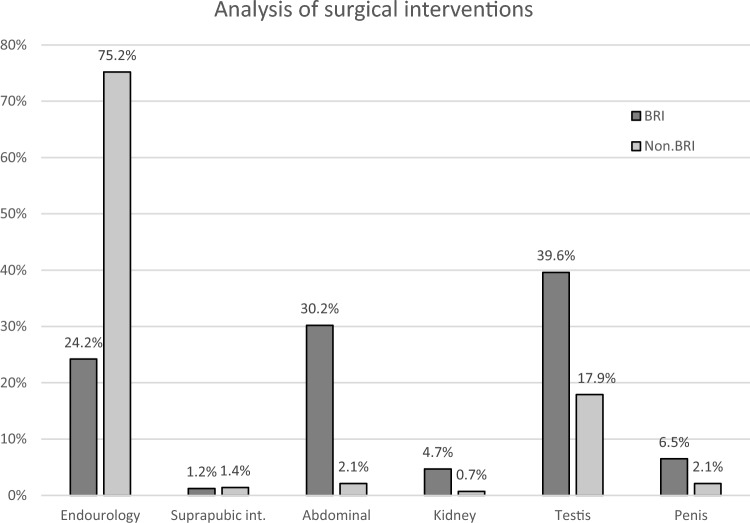
Analysis of surgical urological interventions. In the battle-related intervention group, the prevalence of testis-related interventions prevailed (BRI = 39.6%, n = 67; non-BRI = 17.9%, n = 26, p < 0.001), while in the nonbattle-related intervention group, most interventions were endourological procedures (non-BRI = 75.2%, n = 109; BRI = 24.2%, n = 41, p < 0.001). Furthermore, a significantly higher rate of abdominal interventions was observed in the battle-related intervention group (BRI = 30.2%, n = 51, non-BRI = 2.1%, n = 3, p < 0.001)

Surgical interventions were categorized into battle-related interventions (BRIs, n = 169, 53.8%) and nonbattle-related interventions (non-BRIs, n = 145, 46.2%) (Fig. 2). In the BRI group, interventions were mainly performed on the external genitalia (n = 67, 39.6%, non-BRI: n = 26, 17.9%, p < 0.001), while in the non-BRI group, endourological procedures predominated (non-BRI: n = 109, 75.2%; BRI: n = 41, 24.2%, p < 0.001). This is consistent with a higher rate of laparotomy, abdomen or pelvis procedures performed in the BRI group (n = 51, 30.2%; non-BRI: n = 3, 2.1%, p < 0.001) (Fig. 3).

**Fig. 4:**
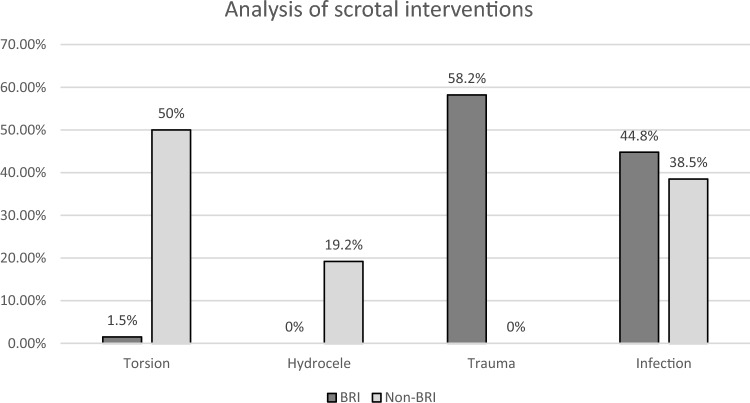
Analysis of scrotal interventions (n = 93). In the battle-related intervention group, most procedures were performed due to scrotal trauma (BRI = 58.2%, n = 39, non-BRI = 0%, p < 0.001), while in the nonbattle-related intervention group, procedures were mainly due to suspected torsion of the testis (non-BRI = 50%, n = 13, BRI = 1.5%, n = 1, p < 0.001) or reduction of hydrocele (non-BRI = 19%, n = 5, BRI = 0%, p < 0.001)

Furthermore, the type of interventions at the external genitalia also differed significantly (Fig. 4). While in the BRI group, 58.2% (n = 39) of interventions were scrotal explorations with either repairs or ablations, none of these procedures were performed in the non-BRI group (p < 0.001). However, 50.0% (n = 13) of scrotal explorations in the non-BRI group were due to suspected torsions of the testis followed by orchidopexy (BRI: n = 1, 1.5%, p < 0.001). Additionally, concerning endourological procedures, there was a significant difference in the use of retrograde urethra/cystograms (RUG). While in the BRI group, the rate was 31.7%, most likely due to suspected injuries of the urethra, in the non-BRI group, the rate of RUGs was only 2.8% (p < 0.001). There were no other significant differences in the distribution of endourological procedures between the BRI and non-BRI groups (cystoscopy, ureterorenoscopy, ureteral stent placement or percutaneous nephrostomy, data not shown).

## Discussion

This study presents the caseload of a deployed German urological surgeon in a U.S. facility (Role 3) in Afghanistan. In terms of urological outpatient care, most consultations in the deployed setting were due to complaints concerning the external genitalia. In contrast, urologists in outpatient care in Germany are mostly consulted due to complaints concerning the prostate (benign prostate hyperplasia or prostate cancer). Obviously, this observation is caused by a younger patient cohort going on deployment, whereas in Germany, most urological outpatients are between 50 and 80 years old [12]. Complaints concerning external genitalia, such as epididymitis, torsion or malignancies of the testes, are primarily problems of younger men [13]. This is consistent with our results regarding distribution of disease patterns in Germany. After excluding patients with complaints not occurring on deployment (pre/aftercare, erectile dysfunction, testosterone substitution, prostate cancer, and renal cyst follow-up), most of the remaining patients presented with complaints concerning the external genitalia and voiding disorders mainly caused by benign prostate hyperplasia. The higher rate of external genitalia complaints could be explained by the fact that our cohort mainly consisted of soldiers aged between 20 and 60 years, which is younger than the typical urological patient cohort in Germany. Further frequent reasons for urological consultations on deployment, such as urolithiasis, infections, hematuria and voiding disorders, coincide with the most frequent consultations domestically in Germany, as our data confirm [12, 14]. Newly diagnosed malignancies are rare because soldiers are subject to precise screening examinations prior to deployment. However, a low percentage of malignancies was uncovered during deployment and leading to repatriation. Therefore, urologists must be aware that urological tumors also occur on assignments abroad and at a higher frequency than in other disciplines.

Furthermore, we analyzed the surgical workload of urological surgeons in Afghanistan by dividing interventions into BRIs and non-BRIs. During recent decades, due to the improvement of Kevlar body armor, the mortality of combat-related injuries has decreased, but the occurrence of genitourinary injuries has increased mainly due to changes in the practice of modern warfare and the lack of sufficient protection. Janak et al. reported that in 12 years (October 2001 – August 2013) of war in Afghanistan and Iraq, there were an unprecedented number (n = 1367) of U.S. American service members with GUIs, confirming the recent increase in GUIs [3]. After battle-related genitourinary injuries, sexual dysfunction, urinary retention/incontinence, urinary tract infections and posttraumatic stress disorder are common complications [15, 16]. To decrease complications, recommendations on the best clinical treatment are needed. While non-BRIs resemble interventions performed domestically, BRIs require additional experience in abdominal/retroperitoneal surgical skills that can be trained in large tumor and basic reconstructive surgeries because of the significant extent of tissue damage typically associated with BRIs [17–20]. Prevention, management and long-term aftercare of genitourinary injuries remain a challenging topic for urological surgeons, and training prior to deployment should intend to optimally prepare them for expected challenges [21]. Training of German urological surgeons in the military is based on civilian residency, and despite special training in emergency medicine, there are no additional contents in reconstructive surgery thus far. In the case of a mass casualty, urologists are part of the emergency surgical team; therefore, they also need to be trained and well prepared.

A limitation of this study methodology that influenced the consistency of the data is that one year of collected data is missing. Nevertheless, the data are homogenous, and there is no evidence that the missing data influence the interpretation of the findings from our research.

In summary, the outpatient care of deployed urological surgeons coincides with domestic outpatient care. In contrast, concerning the care of BRIs, the spectrum of surgical procedures differs from the common workload in Germany, and eventually, further surgical training of urologists in preparation for deployment is recommended.

## Conclusion

Deployed urological surgeons are challenged by a broad spectrum of genitourinary injuries and require additional skills in reconstructive urological surgery compared to those required for daily practice in Germany. Specific surgical training of urological surgeons is warranted to address these issues prior to deployment.

## Supplementary Information

Below is the link to the electronic supplementary material.

Supplementary file1 (DOCX 42 kb)

## Data Availability

The data that support the findings of this study are available from the corresponding author upon reasonable request.
