# Supplementary material for: Urology during Afghanistan mission: lessons learned and implications for the future
Source: World J Urol. 2023 Jun 23;41(8):2195–200. doi: 10.1007/s00345-023-04475-z (PMC10415492; doi:10.1007/s00345-023-04475-z)
Supplement: Supplementary file 1 — Supplementary file1 (DOCX 42 kb) [file 345_2023_4475_MOESM1_ESM.docx]

**Supplementary**

**
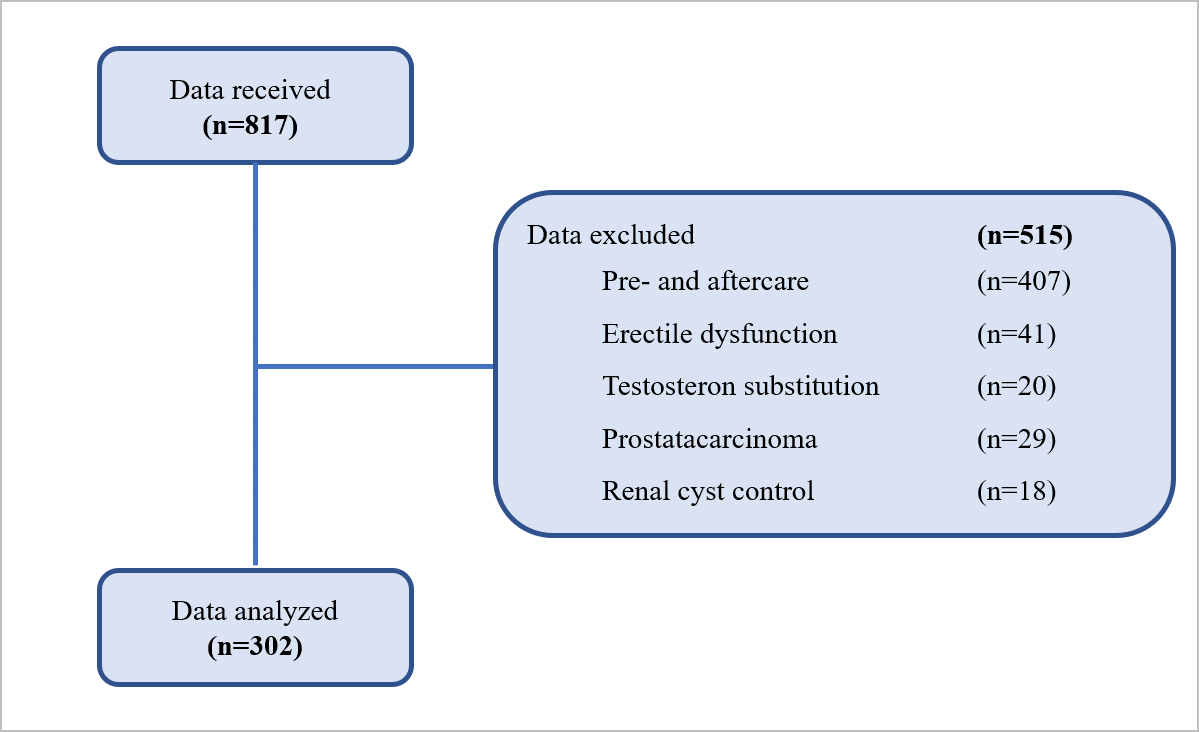
**

**Supp. Fig 1. Domestic outpatient consultations.** Data received from 720 patients with 817 diagnoses and reconsultations were not included. Further data were excluded from the analysis due to the absence of disease patterns on deployment.

**Supp. Fig. 2. Domestical outpatient consultations without reconsultations.** *External genitalia* includes pain or swelling of the external genitalia due to hydro/varicocele or hernia and meatus stenosis. OR procedures were excluded. Consultations of one patient could contain more than one diagnosis.
